# Frequent Hepatitis E Virus Contamination in Food Containing Raw Pork Liver, France

**DOI:** 10.3201/eid2011.140891

**Published:** 2014-11

**Authors:** Nicole Pavio, Thiziri Merbah, Anne Thébault

**Affiliations:** French Agency for Food, Environmental and Occupational Health and Safety, Maisons-Alfort, France

**Keywords:** Hepatitis E virus, foodborne zoonosis, raw pork liver, viruses, France

## Abstract

Food products containing raw pork liver are suspected to be vehicles for transmission of hepatitis E virus. Four categories of food products, comprising 394 samples, were analyzed to determine hepatitis E virus prevalence. Virus was detected in 3%–30% of the different categories. Phylogenetic analysis showed high identity with human and swine sequences.

In humans, hepatitis E virus (HEV) is responsible for an acute, entero-transmissible form of hepatitis, similar to that caused by hepatitis A. In most cases, it is a self-limited infection with rapid viral clearance, but it can evolve into more severe forms, including fatal fulminant hepatitis. Chronic hepatitis E also has occurred in solid-organ transplant recipients and has progressed to more serious conditions, such as fibrosis or liver cirrhosis and liver failure (*1*).

HEV is the only hepatitis virus that can infect species other than primates. HEV infects many animal species, especially pigs, in which a very high prevalence has been described (*2*). Infections acquired in Western countries involve strains that are genetically similar to local swine strains, suggesting an autochthonous origin. Although water is the main vector of contamination in countries to which HEV is endemic, the origin of sporadic cases in other areas is more likely zoonotic. Direct contact with infected animals and consumption of infected meat are possible transmission pathways (*2*).

In France, the annual number of autochthonous cases appears to have increased, from 9 cases in 2002 to nearly 800 in 2012 (http://www.cnrvha-vhe.org/wp-content/uploads/2012/03/2012-Rapport-VHA-VHE.pdf). In a national survey in 2009, the presence of HEV in the swine reservoir was characterized and 65% of pig farms were found to have infected animals; 4% of pork livers entering the food chain were contaminated by the virus (*3*). Molecular analysis of HEV sequences in humans and pigs has shown high identity between the 2 populations (*4*). Food products containing pork liver have repeatedly been suspected of causing indigenous cases of HEV infection (*5*,*6*) and might be responsible for nearly 40% of the autochthonous HEV cases (*12*). Recent studies have confirmed the presence of HEV in the pork food chain, as well as in sausages (*7*,*8*). The objective of this study was to determine the apparent prevalence of HEV contamination in food products containing raw pork liver that were not marketed to be eaten without cooking.

## The Study

In 2011, four different categories of food products in France that were marketed by the food industry were identified as containing raw pork liver out sold to consumers to be eaten after cooking. These 4 categories were 1) figatellu and fitone, 2) dried salted liver, 3) quenelle and quenelle paste, and 4) dried or fresh liver sausages. HEV can be heat-inactivated by thorough cooking at 71°C for 20 min (*9*); however, consumers might not apply such precise thermal treatment. Thus, these food products might be able to transmit HEV. All 4 categories were local regional culinary specialties from eastern or southeastern France. The samples were collected, then frozen directly at the production step after packaging, just before distribution for commercial sale. The frozen samples were sent to the French Agency for Food, Environmental and Occupational Health and Safety laboratory and kept at −80°C until analysis. For each sample, HEV detection was performed on 20 g of product, which had been manually defatted and homogenized in 25 mL phosphate-buffered saline by using a blender. To avoid cross-contamination, each blender was autoclaved between samples. RNA extraction was performed on 500 μL suspension by using the RNeasy lipid Tissue Midi kit (QIAGEN, Hilden, Germany). Presence of inhibitors was assessed by addition of synthetic HEV RNA to the extracts (*9*).

Forty producers were randomly selected, and their relative contributions to total production within each food category were obtained. For each producer, 10 products were randomly selected. Six products were not included in the final analysis because the food category was uncertain. HEV RNA was detected by using real-time reverse transcription PCR as previously described (*9*). Of the 394 food samples analyzed, 68 were found positive (29 figatelli, 1 dried salted liver, 10 quenelle and quenelle paste, and 28 dried or fresh liver sausages). The HEV RNA quantification obtained was 10^2^–10^6^ copies of HEV RNA/g of food (Table). The prevalence for each food category was estimated by using the relative production weight for each food category, and the 95% CI was estimated by bootstrap. Statistical estimates were performed by using R 2.13.1 software (http://www.r-project.org). HEV RNA prevalence was high in figatelli (30% [95% CI 23%–38%]), liver sausages (29% [95% CI 22%–36%]), and quenelles (25% [95% CI 15%–37%]). The prevalence of HEV RNA was lower in dried salted liver: 3% (95% CI 0%–10%) (Table).

**Table:** Quantification and prevalence of HEV RNA in food containing raw pork liver, France, 2011

Food category	No. samples analyzed, N = 394	No. copies HEV RNA/g, range*	Prevalence (95% CI)
Figatelli and fitone	140	1.7 × 10^2^ to 6.9 × 10^5^	0.3 (0.23–0.38)
Dried salted liver	30	6.9 × 10^5^	0.03 (0–0.10)
Quenelle and quenelle paste	55	2.6 v 10^2^ to 2.83 × 10^5^	0.25 (0.15–0.37)
Dried or fresh liver sausages	169	1 × 10^2^ to 2.3 × 10^6^	0.29 (0.22–0.36)

*Minimum and maximum numbers in each food product per category. Detection limit of the method used is 1 × 10^2^ copies of HEV RNA/g. HEV, hepatitis E virus.

Four HEV RNA–positive food samples collected in the present study were tested for infectious virus in collaboration with 2 laboratories (Animal Health and Veterinary Laboratories Agency, Weybridge, UK, and Wageningen University and Research Centre Central Veterinary Institute, Lelystad, the Netherlands). Virus growth from 1 HEV-positive sample was observed in a 3-dimensional culture system developed by these laboratories (*10*). This analysis thus confirms that live viruses can be present in food products.

In preparation of these food products, a large quantity of liver (up to 750 livers per batch) was mixed with fat and spices. Therefore, even if only 4% of raw livers are infected, as shown in a previous study (*3*), the entire batch becomes contaminated; consequently, HEV prevalence is high in the food products. Because high quantities of virus can be present in liver (up to 10^8^ copies of HEV RNA/g) (*9*), the dilution within a large batch will be limited and will not substantially reduce the risk for contamination. The oral infectious dose of HEV is still unknown. In contrast, dried liver is made from only 1 liver; thus the prevalence observed agrees with the prevalence of HEV in liver at the slaughterhouse (i.e., 4%) (*3*).

For further evaluating the link between HEV RNA in pork liver sausages and human autochthonous cases, 68 partial open reading frame 2 sequences were amplified (*3*). This sequence, although short (≈290 nt), reflects the diversity of the HEV full-length genome and is frequently used in phylogenic studies (*11*). All sequences obtained (GenBank accession nos. KJ558436–KJ558503) were of genotype 3, which is the major HEV genotype circulating in autochthonous cases in France and the rest of Europe. The overall mean distance of the sequences from the 68 food products was estimated to be 0.16 nt. To screen for high sequence identity between food products and human or swine HEV, each sequence was analyzed by using BLAST (http://blast.ncbi.nlm.gov/Blast.cgi) to identify the closest sequences. Thirty-three sequences had >98% nt identity with human and/or swine sequences. The human sequences with the highest identity originated in France, except for 2 sequences from the United Kingdom and 1 from Spain (Figure). This result confirms that most autochthonous cases might have a foodborne origin. Two food sequences had >99% nt identity with swine sequences previously described (Figure). Therefore, swine are also sources of autochthonous cases through foodborne transmission.

**Figure:**
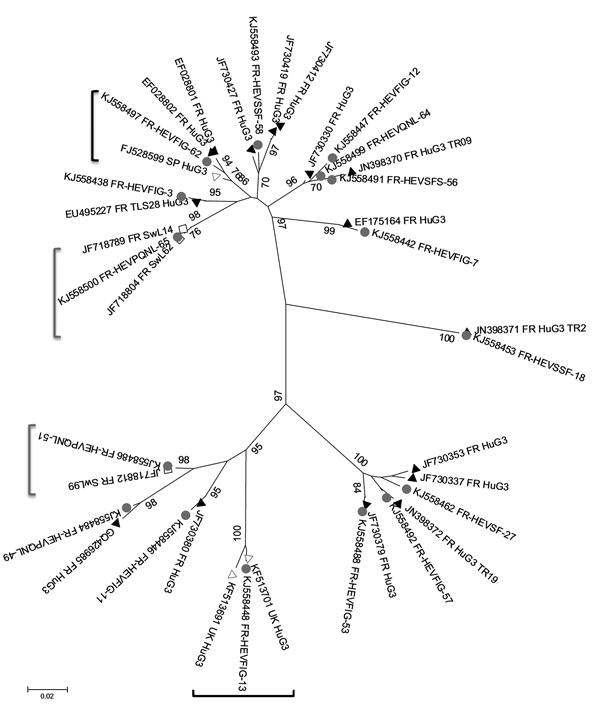
Phylogenetic tree of hepatitis E virus (HEV) sequences identified in food samples, France, 2011. Phylogenetic tree including 16 HEV sequences detected in food samples (gray circles) and the closest human (black triangles, French origin; white triangles, British or Spanish origin) or swine (white squares) sequences was constructed by using the neighbor-joining method with a bootstrap of 1,000 replicates based on the ClustalW alignment (MEGA4, http://www.megasoftware.net) on 290 nt sequences from open reading frame 2. HEV sequences retrieved from GenBank with >98% nt identity are indicated with their accession numbers. Bootstrap values of >70% are indicated on respective branches. Scale bar indicates nucleotide substitutions per site. Similar human and food sequences are shown in black brackets, similar swine and food sequences are shown in gray brackets.

## Conclusions

Our findings clearly demonstrate that some food products that contain raw pork liver and are marketed to be cooked by the consumer can harbor HEV. The close sequence identity observed strongly suggests that foodborne transmission of HEV occurs frequently. Considering this high prevalence, consumers at risk for developing severe forms of HEV (e.g., solid-organ transplant recipients, person having underlying liver conditions, or pregnant women) should be informed about the HEV risk and should avoid eating such pork liver food products without thoroughly cooking them.
